# Synergistic effect of early enteral and parenteral nutrition on immune and nutritional recovery following gastric cancer surgery

**DOI:** 10.3389/fsurg.2025.1679918

**Published:** 2026-01-06

**Authors:** Jialong Tao, Xiuluan Du, Haixia Xu, Liya Dai, Chen Zhang, Wenwen Gao, Sijia Huang, Yanjie Wang, Jing Sun, Wenlu Zhao

**Affiliations:** 1 Department of Oncology, The Second Affiliated Hospital of Soochow University, Suzhou, Jiangsu, China; 2 Department of Pathology, Suzhou Hospital, Affiliated Hospital of Medical School Nanjing University, Suzhou, Jiangsu, China; 3 Department of Traditional Chinese Medicine, The Zhucheng People Hospital, Zhucheng, Shandong, China; 4 Department of Radiology, Suzhou Xiangcheng People’s Hospital, Suzhou, Jiangsu, China; 5 Department of Radiology, The Second Affiliated Hospital of Soochow University, Suzhou, Jiangsu, China

**Keywords:** gastric cancer, radical gastrectomy, early enteral nutrition, parenteral nutrition, immune function

## Abstract

**Objective:**

In this study, we aimed to explore the effects of early enteral nutrition (EN) combined with parenteral nutrition (PN) on the immune function and nutritional indices in patients with gastric cancer (GC) after surgery.

**Methods:**

A total of 100 patients who underwent radical gastrectomy in our hospital between June 2022 and June 2023 were selected and divided into control (CG) and observation (OG) groups. The patients in the CG received early EN support, whereas those in the OG received early EN combined with supportive PN treatment. Gastrointestinal functional recovery, length of hospital stay, nutritional indices, immune function, inflammatory stress index, and the incidence of complications in both groups were compared.

**Results:**

The time to first flatus, time to first defecation, time to tolerance of semi-liquid diet, and the length of hospital stay were shorter in the OG than that in the CG (*P* < 0.05). After nutritional support, the albumin, total protein (TP), and transferrin levels in the OG were higher than those in the CG (*P* < 0.05). In addition, immunoglobulin (Ig)G, IgM, and IgA levels were higher in the OG group than those in the CG group (*P* < 0.05). Moreover, tumor necrosis factor-α, IL-6, and C-reactive protein levels in the OG were lower compared to those in the CG (*P* < 0.05). The incidence of complications was lower in the OG than in the CG (*P* < 0.05).

**Conclusion:**

For patients with GC, the combined application of early EN and PN support measures after surgery can accelerate the recovery of gastrointestinal function, reduce complications, improve the body's nutritional status, promote the recovery of immune function, and lower the inflammatory stress response.

## 1 Introduction

Gastric cancer (GC) is a common type of gastrointestinal cancer characterized by strong invasiveness and poor prognosis (1). The early clinical symptoms of GC are relatively non-specific, mainly manifesting as abdominal distension, dull pain in the upper abdomen, and loss of appetite (2). As the disease progresses, symptoms such as blood disorders, digestive disorders, and in severe cases, pyloric obstruction, bleeding, and life-threatening perforation may occur (3). Radical gastrectomy is a commonly used surgical method in the clinical treatment of GC, with significant effects (4). However, as the surgery is an invasive procedure and a source of stress, it may stimulate pain in the patient, triggering a stress response, leading to excessive catabolic metabolism and malnutrition, thereby having a negative impact on prognosis. Previous studies have shown that active nutritional support after radical surgery can shorten the recovery process (5).

The selection of postoperative nutritional support is still controversial (6). Some studies have shown that early enteral nutrition (EN) can accelerate the absorption of nutrients and help shorten the recovery period (7). However, other studies have indicated that parenteral nutrition (PN) can correct fluid loss and maintain electrolyte balance (8). Nevertheless, both complete EN and PN have certain limitations, particularly in elderly patients (9, 10). Therefore, many studies have proposed that combining PN and EN might provide nutritional support for patients after gastric cancer surgery (11). Hence, in this study, we aimed to assess the effects of early EN combined with PN in patients with GC after surgery.

## 2 Material and methods

### 2.1 General characteristics of patients

A total of 100 patients who underwent radical gastrectomy in our hospital from June 2022 to June 2023 were included in this study. The inclusion criteria were as follows: (1) patients diagnosed with GC using fibroscopy, abdominal ultrasound, and spiral computed tomography (CT), (2) patients with upper abdominal pain, lack of appetite, weight loss, and other clinical symptoms, (3) patients who met the diagnostic criteria for GC, (4) patients who underwent radical gastrectomy, (5) patients with symptoms of malnutrition, (6) patients with complete clinical data, and (7) patients who signed informed consents. The exclusion criteria were as follows: (1) surgical contraindications, (2) other vital organ dysfunction, (3) liver and kidney insufficiency, (4) mental illness, (5) communication disabilities, (6) other malignant tumors, and (7) poor compliance. The patients were divided into a control group (CG) and an observation group (OG), according to the random number table method, with 50 patients in each group. There was no significant difference in the baseline data between the two groups (*P* > 0.05, Table 1). All patients and their families signed the informed consent, and this study was approved by the Ethics Committee of The Second Affiliated Hospital of Soochow University, with the approval number of SZ00-202206[L].

**Table 1:** General characteristics of patients in both groups.

Index		Control group (*n* = 50)	Observation group (*n* = 50)	*P*-value
Gender (male/female)		30/20	29/21	>0.05
Age (years)		51.75 ± 7.45	51.82 ± 7.56	>0.05
Tumor stage	Stage I	17	18	>0.05
	Stage II	25	26	
	Stage III	8	6	
Tumor site	Gastroesophagus	18	19	>0.05
	Body of stomach	15	14	
	Antrum of stomach	17	17	

### 2.2 Randomization and blinding

This study employed a strict randomization grouping method to avoid selection bias. The specific implementation process was as follows: First, a random number table was generated, which was created by professional statisticians using statistical software (such as SAS version 9.4). This random number table ensured the unpredictability and uniform distribution of the random numbers. Then, the patients were numbered sequentially, from 1 to 100, based on their admission order. Next, according to the numbers in the random number table, the patients were assigned to the CG and the OG, with 50 patients in each group. During the entire randomization allocation process, allocation concealment measures were implemented. The random number table and grouping information were kept by independent researchers who were not involved in patient recruitment and the subsequent research implementation. The independent researchers would inform the research implementers of the patients’ groupings based on the random number table only after the patients completed all baseline assessments and met the inclusion criteria, thereby minimizing selection bias to the greatest extent.

This study was conducted using a single-blind method. Due to the significant differences in the implementation methods of EN and PN, as well as the appearance and smell of the nutritional preparations, it was difficult to blind both the researchers and the patients simultaneously. Therefore, only the patients were blinded. Before the start of the study, the researchers explained the research purpose, process, and possible nutritional support methods to the patients in detail but did not inform them of which group they would be assigned to. Throughout the entire study, the patients were unaware whether they were receiving simple early EN or early EN combined with PN, to reduce the influence of psychological suggestion or behavioral changes caused by knowing the group assignment on the research results. Meanwhile, the researchers were aware of the patients’ group assignments so as to accurately implement the corresponding nutritional support plans and collect data.

### 2.3 Sample size calculation

The sample size was calculated based on serum IgG concentration (12). With a statistical power of 80% and a type I error of 2.5%, the number of eligible patients required for this study was calculated to be 45. By considering the 10% exit status, the expected sample size was 50 patients.

### 2.4 Methods

All patients received 200 and 500 mL of oral enteral nutrition emulsions (TPF-D, Fresenius Kabivari Pharmaceutical Co., Ltd., Wuxi, Jiangsu Province, China) 2 and 12 h before surgery, respectively, and the gastric and enteral nutrition tubes were indwelled after surgery.

In this study, the calculation of total calorie intake was mainly based on the patient's weight. The target total calorie intake was set at 25–30 kcal/(kg·day) to meet the basic postoperative energy requirements of patients with gastric cancer and promote their physical recovery. During the actual calculation process, appropriate adjustments were made according to the specific conditions of the patients.

The CG received early EN support and was administered 500 mL of sodium chloride solution (Anhui Shuanghe Pharmaceutical Co., Ltd., Fuyang, Anhai Province, China) within 24 h after the surgery (on the 1st day after surgery). On the 2nd day after surgery, 500 mL of enteral nutrition emulsion (TPF-D) was administered; at first the infusion rate was controlled at 20 mL/h, and if the patient tolerated it, the infusion rate was gradually increased to 30 mL/h. On the third day after surgery, 500 mL of enteral nutrition emulsion (TPF-D) was administered at a rate of 40 mL/h, and if the patient tolerated it, the rate was gradually increased to 50 mL/h. On the fourth to seventh day after surgery, 500–1,000 mL of enteral nutrition emulsion (TPF-D) was administered, and the drip rate was controlled at 60–100 mL/h.

Patients in the OG received early EN combined with supportive PN treatment. On the first day after surgery, a total intravenous nutritional solution [fat milk (10%)/amino acid (15%)/glucose (20%) injection; Sichuan Kelon Pharmaceutical Co., Ltd., Chengdu, Sichuan Province, China] was slowly injected, according to the patient's specific conditions, and 500 mL of 0.9% sodium chloride solution was injected using a nasointestinal tube. The speed was 6–13 drops/min, and EN was initiated simultaneously. The total heat was 25 kcal/(kg·day), and the EN amount was reduced according to the PN amount. On the second day after surgery, 500 mL of isotonic saline was injected using the nasointestinal tube. On the third day after surgery, 500 mL of TPF-D was administered, and the drip rate was controlled at 50 mL/h. On the fourth to seventh days after surgery, 25–30 mL/kg TPF-D was injected for 16–19 h, and PN supplementation was administered if intolerance was observed.

Both groups were treated continuously for 7 days.

### 2.5 Measurement outcomes

#### 2.5.1 Primary outcomes

Indices of gastrointestinal functional recovery, including time to first flatus, time to first defecation, and time to tolerance of semi-liquid diet, as well as the length of hospital stay were compared between the two groups.

Nutritional indices: venous blood (4 mL) was collected before and 7 days after surgery and centrifuged at 3,000 rpm for 15 min. The serum was separated, and the levels of transferrin (TRF), prealbumin (PA), and albumin (ALB) were determined using enzyme-linked immunosorbent assay (Shanghai Enzyme-linked Biotechnology Co., Ltd., Shanghai, China).

Immune function indicators: The levels of immunoglobulin G (IgG), immunoglobulin M (IgM), and immunoglobulin A (IgA) in the two groups were measured using immunoturbidimetry (Shanghai Enzyme-linked Biotechnology Co., Ltd., Shanghai, China).

#### 2.5.2 Secondary outcomes

Inflammatory stress indices: The levels of tumor necrosis factor-α (TNF-α), interleukin-6 (IL-6), and C-reactive protein (CRP) were measured by enzyme-linked immunosorbent assay (Beyotime Biotechnology, Shanghai, China).

Complications: The incidence of complications, such as vomiting, nausea, and abdominal distension, were analyzed in the two groups. This study employed a combination of observation records and patients’ self-reports to document the occurrence of complications. The recording period of complications covered the entire period of the patient's hospital stay after the operation. The research team arranged for experienced medical staff to closely monitor the patients. During the patients’ postoperative hospital stay, the medical staff conducted regular rounds every day, carefully examining the patients’ physical conditions, including observing their facial expressions and body movements to determine if there was any discomfort, checking for signs of abdominal distension and tenderness, to assess the degree of abdominal distension, and paying attention to whether the patients had dry vomiting or vomiting actions. At the same time, the medical staff would meticulously record the patients’ vital signs, the nature and volume of gastrointestinal decompression drainage, and other information. In addition to the observation records made by medical staff, we also encouraged patients to actively report their own discomfort symptoms. When patients were admitted to the hospital, medical staff thoroughly explained to the patients and their families the possible symptoms of complications, At the same time, we informed patients that if they experienced any discomfort after the surgery, they should inform the medical staff promptly. To facilitate patients’ reporting, we set up clear indication signs in the ward, and medical staff regularly inquired about patients’ feelings of discomfort.

### 2.6 Statistical analysis

The SPSS 22.0 software was used for statistical analysis of the data. For continuous data, we conducted a normality test. The Shapiro–Wilks test was used to assess whether the data followed a normal distribution. Only when the data met the prerequisite of being normally distributed did we represent the continuous data in the form of mean ± standard deviation and use the *t*-test for inter-group comparisons. If the data did not satisfy the normal distribution, we would use the Mann–Whitney *U*-test for inter-group comparisons. When dealing with repeated measurement data, we employed the paired *t*-test. Categorical data were expressed as the number of cases and rate (%), and *χ*^2^ test was used for comparisons between groups. Statistical significance was set at *P* < 0.05.

## 3 Results

### 3.1 Gastrointestinal functional recovery indices and length of hospital stay

**Figure 1:**
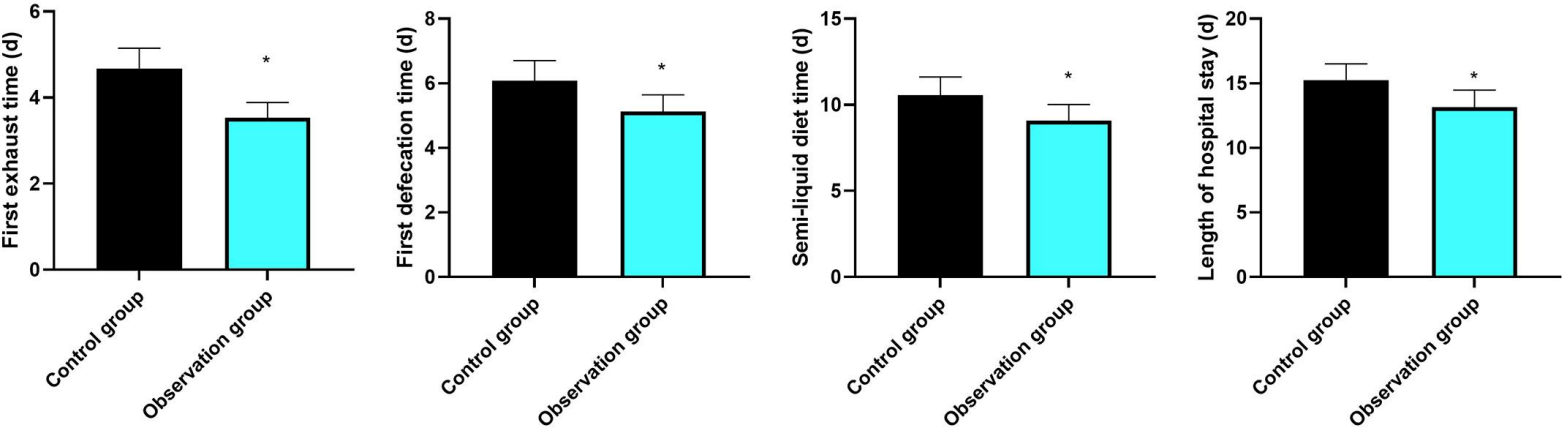
Gastrointestinal functional recovery indices and length of hospital stay. *P < 0.05, compared with the control group.

As shown in Figure 1, the time to first flatus, time to first defecation, time to tolerance of semi-liquid diet, and the length of hospital stay in the CG were 4.68 ± 0.47, 6.08 ± 0.62, 10.56 ± 1.06, and 15.25 ± 1.26 days, and those in the OG were 3.53 ± 0.36, 5.12 ± 0.52, 9.10 ± 0.92, and 13.15 ± 1.32 days, respectively. Compared with the CG, the time to first flatus, time to first defecation, time to tolerance of semi-liquid diet, and the length of hospital stay were significantly shorter in the OG (*P* < 0.05), and the 95% CIs were −1.316 to 0.983, −1.187 to 0.732, and −1.854 to 1.066, respectively.

### 3.2 Nutritional indices

Before nutritional support, the ALB, total protein (TP), and TRF levels in the CG were 25.63 ± 2.56 g/L, 136.52 ± 13.67 mg/L, and 2.12 ± 0.21 g/L, and those in the OG were 25.58 ± 2.59 g/L, 136.48 ± 13.72 mg/L, and 2.13 ± 0.22 g/L, respectively.

After nutritional support, the ALB, TP, and TRF levels in the CG were 33.54 ± 3.36 g/L, 145.63 ± 14.57 mg/L, and 2.40 ± 0.24 g/L, and those in the OG were 37.68 ± 3.78 g/L, 165.87 ± 16.68 mg/L, and 2.62 ± 0.27 g/L, respectively.

There were no differences in ALB, TP, and TRF levels between the two groups before nutritional support (*P* > 0.05), and the 95% CIs were −1.027 to 0.927, −5.239 to 5.159, and −0.071 to 0.091, respectively.

After nutritional support, the ALB, TP, and TRF levels increased in both groups (*P* < 0.05), and the 95% CIs were 6.723–9.097, 3.503–14.72, and 0.190–0.369 in the CG and 10.93–13.27, 23.87–34.91, and 0.400–0.579 in the OG, respectively.

**Figure 2:**
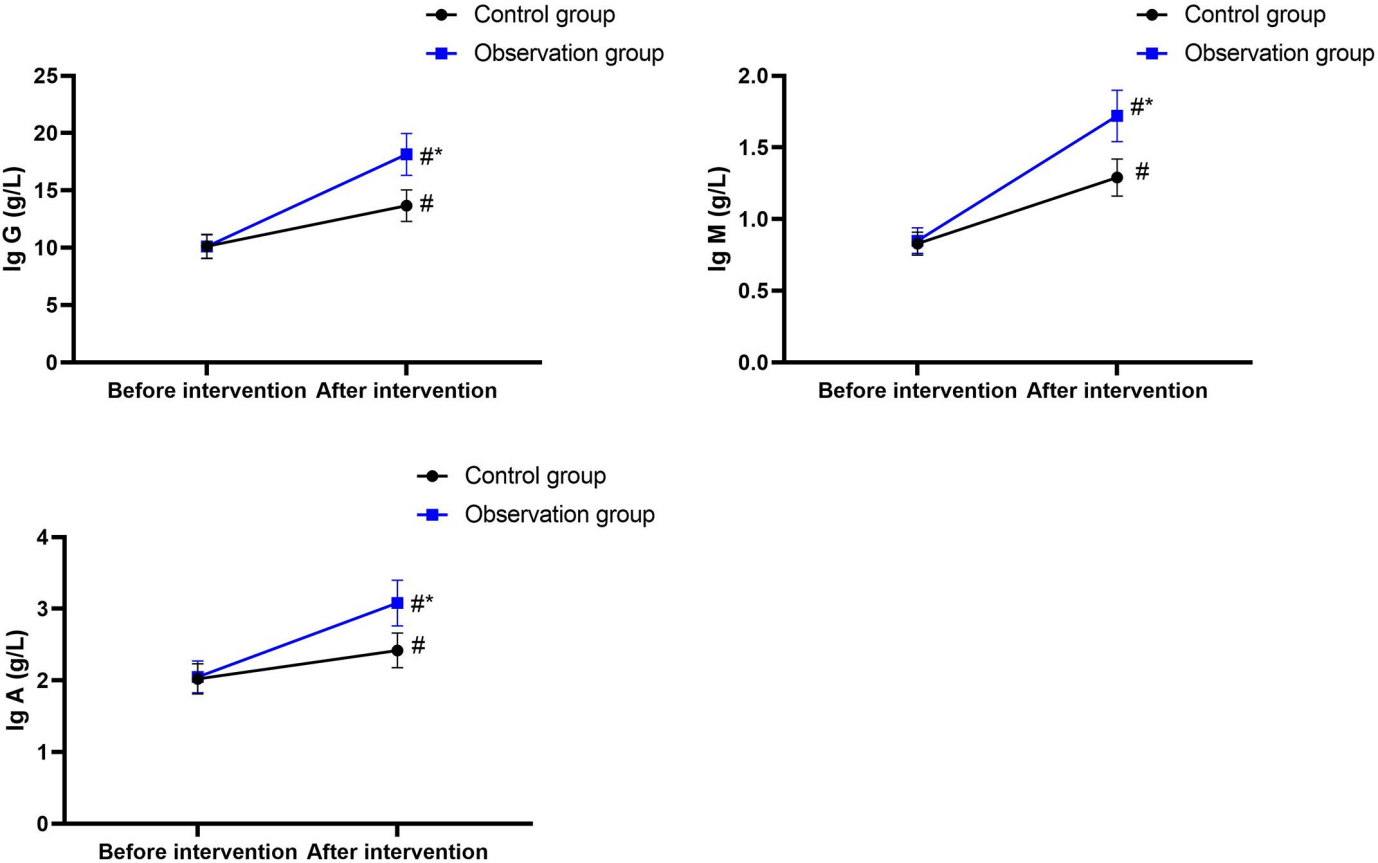
Nutritional indices. ^#^*P* < 0.05, compared with before intervention. *P < 0.05, compared with the control group.

After nutritional support, the ALB, TP, and TRF levels in the OG were higher than those in the CG (*P* < 0.05), and the 95% CIs were 2.790–5.490, 14.33–26.15, and 0.123–0.316, respectively (Figure 2).

### 3.3 Immune function indicators

Before nutritional support, the IgG, IgM, and IgA levels in the CG were 10.13 ± 1.05 g/L, 0.83 ± 0.08 g/L, and 2.02 ± 0.21 g/L, and those in the OG were 10.12 ± 1.02 g/L, 0.85 ± 0.09 g/L, and 2.05 ± 0.22 g/L, respectively.

After nutritional support, the IgG, IgM, and IgA levels in the CG were 13.68 ± 1.37 g/L, 1.29 ± 0.13 g/L, and 2.42 ± 0.24 g/L, and those in the OG were 18.15 ± 1.82 g/L, 1.72 ± 0.18 g/L, and 3.08 ± 0.32 g/L, respectively.

There were no differences in IgG, IgM, and IgA levels between the two groups before nutritional support (*P* > 0.05), and the 95% CIs were −0.420 to 0.400, −0.012 to 0.053, and −0.055 to 0.115, respectively.

After nutritional support, the IgG, IgM, and IgA levels increased in both groups (*P* < 0.05), and the 95% CIs were 3.065–4.035, 0.417–0.502, and 0.310–0.489 in the CG and 7.442–8.618, 0.813–0.926, and 0.920–1.139 in the OG, respectively.

**Figure 3:**
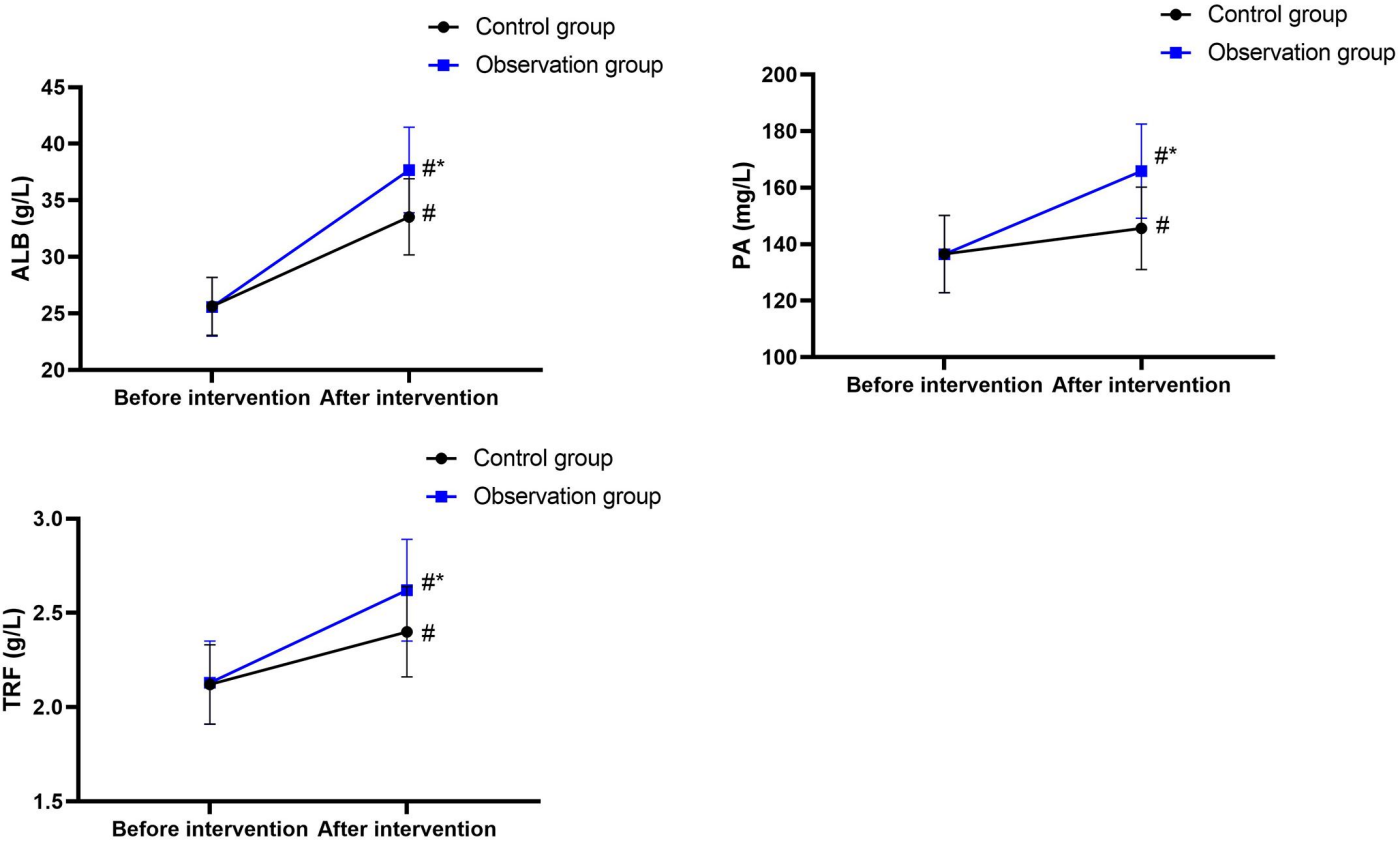
Immune function indicators. ^#^*P* < 0.05, compared with before intervention. *P < 0.05, compared with the control group.

After nutritional support, IgG, IgM, and IgA levels increased in both groups, with higher values in the OG than those in the CG (*P* < 0.05), and the 95% CIs were 3.830–5.110, 0.369–0.490, and 0.547–0.772, respectively (Figure 3).

### 3.4 Inflammatory stress index

Before nutritional support, the TNF-α, IL-6, and CRP levels in the CG were 175.16 ± 17.26 ng/L, 235.75 ± 23.76 ng/L, and 156.65 ± 15.64 mg/L, and those in the OG were 175.23 ± 17.28 ng/L, 235.78 ± 23.69 ng/L and 158.74 ± 15.84 mg/L, respectively.

After nutritional support, the TNF-α, IL-6, and CRP levels in the CG were 233.89 ± 23.46 ng/L, 440.17 ± 44.09 ng/L, and 235.68 ± 23.56 mg/L, and those in the OG were 205.49 ± 20.49 ng/L, 340.39 ± 34.41 ng/L, and 193.98 ± 19.87 mg/L, respectively.

There was no difference in TNF-α, IL-6, and CRP levels between the two groups before nutritional support (*P* > 0.05), and the 95% CIs were −6.784 to 6.924, −9.386 to 9.446, and −4.157 to 8.337, respectively.

After nutritional support, the TNF-α, IL-6, and CRP levels increased in both groups (*P* < 0.05), and the 95% CIs were 50.55–66.91, 190.3–218.5, and 71.08–86.98 in the CG and 22.73–37.79, 92.87–116.4, and 28.10–42.38 in the OG, respectively.

**Figure 4:**
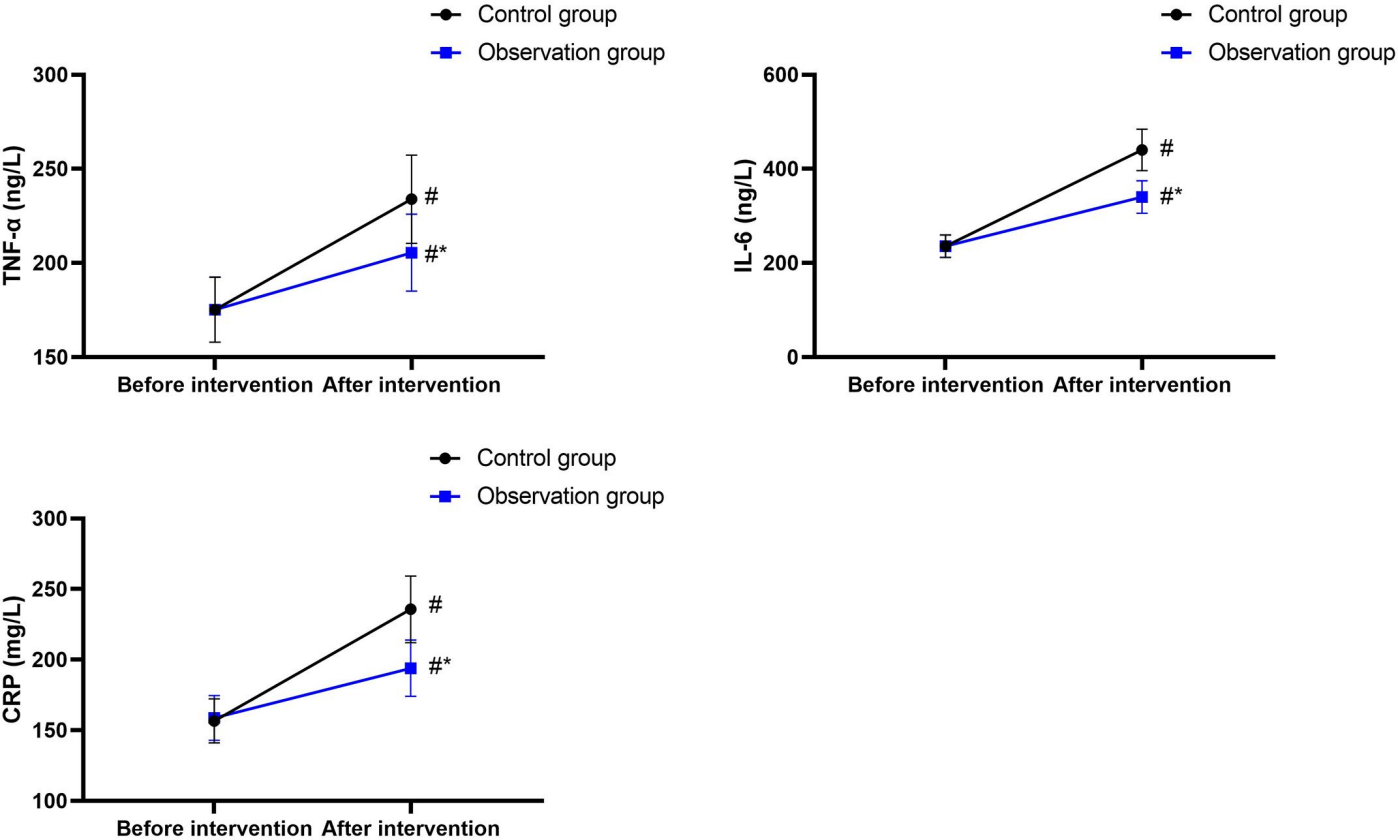
Inflammatory stress index. ^#^*P* < 0.05, compared with before intervention. *P < 0.05, compared with the control group.

After nutritional support, the TNF-α, IL-6, and CRP levels were lower in the OG than those in the CG (*P* < 0.05), and the 95% CIs were −37.14 to 19.66, −115.5 to 84.07, and −50.35 to 33.05, respectively (Figure 4).

### 3.5 Incidence of complications

In addition, the incidence of complications was lower in the OG than that in the CG (Table 2, *P* < 0.05, 95% CI: 0.094–0.910).

**Table 2:** Incidence of complications in both groups.

Groups	*n*	Vomiting	Nausea	Abdominal distension	Total incidence rate
Observation group	50	1	1	0	2 (4%)
Control group	50	3	3	3	9 (18%)
*χ* ^2^					5.005
*P-*value					0.025

## 4 Discussion

GC is usually accompanied by digestive tract obstruction, which has a negative impact on the patient's ability to eat (13). Moreover, the tumor itself may also cause malnutrition; therefore, patients with GC may be in a state of malnutrition before surgery, resulting in immune dysfunction and an increased risk of postoperative incision infection (14).

Radical gastrectomy can effectively remove the tumor, alleviate clinical symptoms, and promote the recovery of patients (15). However, considering that patients with GC have a weakened immune system and the invasiveness of the surgery, patients may experience a stress response, which can result in postoperative malnutrition, affecting postoperative recovery (16). Therefore, for patients with GC, timely provision of nutritional support is particularly important.

Early EN and PN are two commonly used clinical nutritional support methods. They can effectively improve the gastrointestinal function and nutritional status of patients, and promote the recovery of immune function (17). However, they also have some limitations. For example, the effectiveness of complete EN support largely depends on the volume, concentration, and infusion rate of the nutritional solution, which may result in inadequate feeding or the patient's inability to tolerate it, ultimately leading to gastrointestinal complications such as abdominal distension and nausea (18). In addition, long-term use of total PN may cause internal environment disorders and decline in gastrointestinal function (19).

In our study, the time to first flatus, time to first defecation, time to tolerance of semi-liquid diet, and the length of hospital stay were shorter in the OG than those in the CG. After nutritional support, ALB, TP, and TRF levels in the OG were higher than those in the CG, and IgG, IgM, and IgA levels in the OG were higher than those in the CG. The incidence of complications was lower in the OG than in the CG. These results suggested that early EN combined with PN could accelerate the recovery of gastrointestinal function, shorten the hospital stay, improve nutritional status and immune function, and reduce the incidence of complications in patients with GC after surgery, which was consistent with the findings of a previous study (20). PN is supplemented through nasal feeding, which can directly deliver the nutrient solution to the stomach, thereby accelerating protein synthesis (10). Therefore, early EN may have a synergistic effect, providing patients with nutrient preparations rich in energy and protein, which can effectively inhibit the release of cytokines, stimulate the immune response, and have a relatively small impact on gastrointestinal function, thereby accelerating the repair of gastrointestinal barrier function and improving immune function (21). In addition, the combined application of early EN and PN may reduce the volume of nutrient solution injected in early EN support programs, thereby minimizing the gastrointestinal complications such as abdominal distension, vomiting, and nausea caused by gastrointestinal intolerance to the greatest extent (22).

In addition, IL-6, TNF-α, and CRP are commonly used to evaluate the degree of inflammatory stress in the body (23). IL-6 is a lymphokine secreted by activated fibroblasts and T cells and promotes B-cell proliferation (24). TNF-α is a proinflammatory factor with strong proinflammatory effects (25). CRP is an acute protein secreted in response to tissue damage or microbial invasion and inflammation, where CRP levels reflect the degree of inflammatory stress (26). In our study, after nutritional support, TNF-α, IL-6, and CRP levels in the OG were lower than those in the CG, suggesting that early EN combined with PN could effectively reduce the degree of inflammatory stress response in patients with GC after surgery, which is in line with the results of Mańkowska-Wierzbicka et al. (27). It has been shown that the combined application of EN and PN can promote gastrointestinal peristalsis, accelerate body metabolism, reduce exotoxin and bacterial translocation, effectively alleviate the inflammatory response, and reduce the degree of stress (28).

The results of this study are highly consistent with the guidelines of enhanced recovery after surgery (ERAS) and the current standards for tumor nutrition therapy. The ERAS guidelines emphasize the implementation of a series of optimization measures during the perioperative period to facilitate rapid recovery of patients, with nutrition support being one of the key aspects (29). Early EN can directly provide nutrients to the intestinal mucosa, stimulate intestinal peristalsis and secretion of digestive juices, and maintain the integrity and barrier function of the intestinal mucosa, which is consistent with the concept advocated by the ERAS guidelines of restoring intestinal function as soon as possible and reducing intestinal complications. Parenteral nutrition can provide necessary energy and nutrients when patients cannot tolerate enteral nutrition or when enteral nutrition cannot meet the body's needs, and the combined application of the two can more comprehensively meet the nutritional support needs of patients with gastric cancer after surgery.

The current guidelines for tumor nutrition therapy also clearly state that for patients with malignant tumors, especially those who have undergone surgery, an individualized nutrition support plan should be formulated based on the specific conditions of the patients. Postoperative patients with gastric cancer often have problems such as impaired digestive function and insufficient nutrient intake. The combined early EN and PN support measures can gradually increase the intake of EN based on the patient's gastrointestinal tolerance while also supplementing with PN. This not only avoids the potential nutritional deficiency caused by simple EN but also reduces complications such as intestinal mucosa atrophy and intestinal flora imbalance caused by simple PN, which is in line with the individualized and comprehensive nutrition support principles of tumor nutrition therapy guidelines.

The results of this study have significant translational significance for daily clinical practice. In clinical work, the formulation of postoperative nutritional support plans for patients with gastric cancer has always been a focus of attention for medical staff. The traditional nutritional support methods may be monotonous and cannot fully meet the complex nutritional needs of patients after surgery. However, the early combination of enteral nutrition and parenteral nutrition support measures provides a more scientific and effective nutritional support plan for clinical practice.

By implementing this combined nutritional support measure, medical staff can more accurately assess the patient's nutritional status and the recovery of gastrointestinal function, and promptly adjust the nutritional support plan. For example, in the early postoperative period when the gastrointestinal function has not fully recovered, the proportion of parenteral nutrition can be appropriately increased to ensure the patient's energy and nutrient intake; as the gastrointestinal function gradually recovers, the amount of enteral nutrition can be gradually increased to promote further recovery of intestinal function. This not only helps improve the patient's rehabilitation quality and shorten the hospital stay but also reduces the patient's medical expenses and improves the utilization efficiency of medical resources.

Furthermore, the implementation of such combined nutritional support measures also requires healthcare professionals to possess more comprehensive nutritional knowledge and skills. Therefore, the results of this study also suggest that clinical settings should strengthen the nutritional training for healthcare staff and enhance their understanding of the importance of nutritional support and their ability to implement nutritional support plans, so as to better serve patients.

Regarding whether this treatment method is applicable to other types of abdominal cancers, the current research evidence indicates that it has a certain degree of general applicability. From a physiological perspective, after surgery, patients with abdominal cancers often encounter problems such as impaired gastrointestinal function and metabolic disorders, similar to those of patients with gastric cancer. The early combination of enteral nutrition and parenteral nutrition support measures helps improve the postoperative recovery of patients by promoting the recovery of gastrointestinal function, providing comprehensive nutritional support, and regulating immune function and inflammatory responses.

However, due to the differences in the biological behavior, surgical methods, and tolerance to nutritional support among various abdominal cancers, further large-scale clinical studies are needed when applying this treatment method to other abdominal cancers to clarify its specific application indications, nutritional support plans, and efficacy evaluation criteria. At the same time, individualized nutritional support plans should be developed based on the characteristics of different cancers and the individual conditions of patients to achieve the best therapeutic effect.

Our research has some limitations. First, our sample size is relatively small, which may lead to deviations between the data results and the actual values. Second, our research adopted a single-blind design, which inevitably resulted in subjective biases from the researchers, leading to an imbalance in the treatment between the two groups. Third, our research was a single-center study and the sample was not representative, which may not accurately reflect the characteristics of a broader population. Fourth, our research did not conduct long-term follow-up observations. The effects of early EN and PN support on the long-term prognosis of patients with GC after surgery are currently unclear. Therefore, more multi-center, double-blind, large-scale, and long-term studies should be conducted in the future to further verify our findings.

In conclusion, our study demonstrates that for patients with GC, the combined application of early EN and PN support measures after surgery can accelerate the recovery of gastrointestinal function, reduce complications, improve the body’s nutritional status, promote the recovery of the immune function, and lower the inflammatory stress response.

## Data Availability

The datasets presented in this study can be found in online repositories. The names of the repository/repositories and accession number(s) can be found in the article/Supplementary Material.
